# Plasma AGE and Oxidation Products, Renal Function, and Preeclampsia in Pregnant Women with Type 1 Diabetes: A Prospective Observational Study

**DOI:** 10.1155/2023/8537693

**Published:** 2023-08-10

**Authors:** Clare B. Kelly, Harsha Karanchi, Jeremy Y. Yu, Misti J. Leyva, Alicia J. Jenkins, Alison J. Nankervis, Kristian F. Hanssen, Satish K. Garg, James A. Scardo, Samar M. Hammad, Christopher E. Aston, Paul J. Beisswenger, Timothy J. Lyons

**Affiliations:** ^1^Division of Endocrinology, Medical University of South Carolina, Charleston, South Carolina, USA; ^2^Centre for Public Health, School of Medicine, Dentistry and Biomedical Sciences, Queen's University Belfast, Belfast, UK; ^3^Diabetes Free South Carolina, BlueCross BlueShield of South Carolina, Columbia, South Carolina, USA; ^4^Baker Heart and Diabetes Institute, Melbourne, VIC, Australia; ^5^Diabetes Service, The Royal Women's Hospital, Melbourne, VIC, Australia; ^6^Department of Endocrinology, Oslo University Hospital, Oslo, Norway; ^7^Institute of Clinical Medicine, University of Oslo, Oslo, Norway; ^8^Barbara Davis Center for Childhood Diabetes, University of Colorado, Denver, Colorado, USA; ^9^Spartanburg Regional Medical Center, Spartanburg, South Carolina, USA; ^10^Department of Regenerative Medicine and Cell Biology, Medical University of South Carolina, Charleston, South Carolina, USA; ^11^Department of Pediatrics, University of Oklahoma Health Sciences Center, Oklahoma City, Oklahoma, USA; ^12^PreventAGE Health Care, Lebanon, NH, USA

## Abstract

**Aims:**

We aimed to determine whether plasma advanced glycation end products or oxidation products (AGE/oxidation-P) predict altered renal function and/or preeclampsia (PE) in pregnant women with type 1 diabetes.

**Methods:**

Prospectively, using a nested case-control design, we studied 47 pregnant women with type 1 diabetes, of whom 23 developed PE and 24 did not. Nineteen nondiabetic, normotensive pregnant women provided reference values. In plasma obtained at ~12, 22, and 32 weeks' gestation (visits 1, 2, and 3; V1-V3), we measured five AGE products (carboxymethyllysine (CML), carboxyethyl-lysine (CEL), methylglyoxal-hydroimidazolone (MGH1), 3-deoxyglucosone hydroimidazolone (3DGH), and glyoxal-hydroimidazolone (GH1)) and four oxidation products (methionine sulfoxide (MetSO), 2-aminoadipic acid (2-AAA), 3-nitrotyrosine (3NT), and dityrosine (DT)), by liquid chromatography/mass spectroscopy. Clinical outcomes were “estimated glomerular filtration rate” (eGFR) at each visit and onset of PE.

**Results:**

In diabetic women, associations between AGE/oxidation-P and eGFR were found only in those who developed PE. In this group, CEL, MGH1, and GH1 at V2 and CML, CEL, MGH1, and GH1 at V3 were inversely associated with contemporaneous eGFR, while CEL, MGH1, 3DGH, and GH1 at V2 were inversely associated with eGFR at V3 (all *p* < 0.05). There were no associations of plasma AGE or oxidation-P with pregnancy-related development of proteinuria or PE.

**Conclusions:**

Inverse associations of second and early third trimester plasma AGE with eGFR among type 1 diabetic women who developed PE suggest that these patients constitute a subset susceptible to AGE-mediated injury and thus to cardiorenal complications later in life. However, AGE/oxidation-P did not predict PE in type 1 diabetic women.

## 1. Introduction

Preeclampsia (PE) is a serious hypertensive complication of pregnancy. For poorly understood reasons, it occurs 4-5 times more often in women with than without type 1 diabetes, even in those without clinical evidence of prior vascular complications [1]. PE is associated with future cardiovascular disease (CVD), renal dysfunction, and hypertension [2–4].

Advanced glycation end products (AGE) are implicated in the micro- [5] and macro-vascular [6] complications of diabetes. In the formation of AGE, biomolecules are modified by reactive carbonyl species (e.g., glyoxal, methylglyoxal, and 3-deoxyglucose) formed either by free radical oxidation or in metabolic pathways (e.g., glycolytic pathway) [7]: both these mechanisms are amplified by diabetes. AGE themselves increase oxidative stress and inflammation via interaction with a cell-bound “receptor for AGE” (RAGE), thus promoting vicious circles of injury [8, 9].

Clearance mechanisms for AGE ameliorate their toxicity. Scavenger receptors (e.g., CD36, OST-48, and galectin-3) play an important role via receptor-mediated endocytosis, which is followed by lysosomal degradation, yielding AGE peptides that are cleared by the kidney [10, 11]. The “soluble receptor for AGE” (sRAGE) is formed either by proteolytic cleavage of membrane-bound RAGE [12] or by alternative splicing [13]. It acts as a decoy, sequestering circulating AGE and preventing binding to RAGE [14, 15]. AGE trigger proinflammatory, profibrotic, and procoagulatory cellular responses that are capable of causing tissue injury. Mechanisms implicating the “AGE-RAGE axis” in renal injury associated with diabetes were recently summarized by Sanajou et al. [16].

AGE have adverse effects on the placenta and have been associated with PE [17–22]. Placental hypoxia and resultant oxidative stress are linked with the pathophysiology of preeclampsia, and AGE have been shown to have a role to mediate oxidative stress [8, 9]. AGE may also inhibit trophoblast invasion [20] and increase antiangiogenic soluble fms-like tyrosine kinase (sFlt-1) to a greater extent than pro-angiogenic vascular endothelial growth factor (VEGF), thus diminishing placental angiogenesis [23]. Impaired trophoblast invasion and impaired angiogenesis are both implicated in PE. However, no previous study has systematically investigated whether plasma AGE concentrations early in pregnancy are associated with subsequent renal dysfunction or PE in women with diabetes.

In the “Markers and Mechanisms for PreEclampsia in Type 1 Diabetes” (MAMPED) study, we used a case-control approach and analyzed samples collected at three time-points from the first to the early third trimesters in women with and without PE. In a previous report, we used immunologic techniques to measure two specific AGE, serum N^*ε*^-(carboxymethyl)lysine (CML, a dominant AGE) and hydroimidazolone (methylglyoxal-modified proteins), as well as a global measure of “total AGE” [24]. While these products did not predict subsequent PE, low serum sRAGE did so [24]. Another previous and relevant MAMPED finding was that in the first trimester estimated glomerular filtration rate (eGFR) was associated with subsequent PE occurring late in the third trimester [25].

In this study, we hypothesized that analysis of a larger number of plasma AGE and, in addition, specific oxidation products would reveal associations with renal function (eGFR) and, perhaps, new markers for subsequent PE. In plasma from a predefined MAMPED subset, we used more rigorous methodology, liquid chromatography/mass spectrometry, to measure nine products, including five carbonyl-derived AGE (CML, carboxyethyl-lysine (CEL), glyoxal-hydroimidazolone (GH1), methylglyoxal-hydroimidazolone (MGH1), and 3-deoxyglucosone hydroimidazolone (3DGH)) and four oxidation products (methionine sulfoxide (MetSO), 2-aminoadipic acid (2-AAA), 3-nitrotyrosine (3NT), and dityrosine (DT)). This panel has been shown, in large cohorts of older patients (unrelated to pregnancy) to predict decline in eGFR over 6-12 years [26, 27]. We aimed to determine if any of these products, or their ratios with serum sRAGE (the latter available from preexisting data), were cross-sectionally or prospectively associated with eGFR and/or PE in pregnant women with type 1 diabetes.

## 2. Research Design and Methods

### 2.1. Study Participants

The MAMPED study design, participants, and enrollment criteria have been described previously [28]. Briefly, the study was conducted according to the principles of the Declaration of Helsinki and was approved by the Institutional Review Boards of all six participating institutions in three countries (Australia, Norway, and USA). Written informed consent was obtained from all study participants. Pregnant women with type 1 diabetes (*n* = 151) were enrolled in the first trimester and followed throughout pregnancy. A group of healthy non-diabetic women (*n* = 24) was included to provide reference control values: its size was intended to approximate the number of PE cases among the diabetic women.

At enrollment (average gestational age 12 weeks), all women were normotensive and free of microalbuminuria or overt proteinuria (i.e., urinary albumin-to-creatinine ratios were <30 mg/g). Clinical data and blood and urine specimens were collected at three study visits, 12.2 ± 1.9, 21.6 ± 1.5, and 31.5 ± 1.7 weeks' gestation (mean ± standard deviation (SD)), corresponding to late first, mid-second, and early third trimesters. Importantly, all (including third trimester) study visits took place before the onset of PE. Fasting blood samples were centrifuged promptly, and plasma was stored at -80°C until analysis. PE cases were defined having new-onset hypertension (>140/90 mm Hg) and proteinuria (>300 mg/24 hours) after 20 weeks of gestation in a previously normotensive woman.

MAMPED was designed as a nested case-control study. Consistent with the original data analysis plan and using all samples available, we analyzed plasma samples from 23 of the original 26 diabetic women who developed PE (DM+PE+), and 24 of 26 matched diabetic women who remained normotensive (DM+PE-). Nineteen of 24 normotensive, non-diabetic women served as “reference controls” (DM-). The primary analysis was between the two diabetic groups, which were matched for age, diabetes duration, and parity.

### 2.2. Laboratory Measures

Plasma AGE and oxidation products were measured by liquid chromatography/mass spectrometry with internal stable heavy isotope-substituted standards on an Agilent model 6490 Triple Quadrupole MS System with a 1290 Rapid Resolution LC System for analyte detection. All AGE and oxidation products were separated and analyzed in a single run using a single Waters X-select HSS T3 2.5 *μ*m × 2.1 × 150 mm column, as previously described [26, 27, 29, 30]. Laboratory analyses were performed in a blinded fashion. Briefly, AGE and oxidation products were quantified in plasma filtrates prepared via centrifugation through 10 K Dalton cut-off Amicon filters and separated by liquid chromatography with a methanol/H_2_O gradient mobile phase with 0.29% heptafluorobutyric acid. Approximately 40% of all the samples analyzed had GH1 concentrations below the lower limit of quantitation (LLOQ = 5.4 nmol/L) (no significant difference between women with/without diabetes and/or PE), and for these, a value of 50% of the LLOQ was assigned. Data were analyzed both with and without inclusion of these estimations: significances of differences did not change, and the presented data include the estimates. Plasma levels of 3NT and DT were undetectable in every participant and are neither presented nor discussed. All other AGE and oxidation products were measurable in all samples; all reported values were corrected for plasma total protein concentration.

Serum sRAGE (total circulating pool) was measured, as previously described and reported, and quantified by a DuoSet enzyme-linked immunosorbent assay (ELISA) kit (DY1145) according to the manufacturer's protocol (R&D Systems Inc., Minneapolis, MN, USA) [24].

Serum creatinine was measured and GFR estimated (eGFR) as previously described [25]. All women had eGFR > 60 mL/min/1.73 m^2^ throughout pregnancy and thus were classified as having normal renal function.

### 2.3. Statistical Analysis

As predefined in MAMPED, we studied matched subsets (matched for age, diabetes duration, and parity) with and without PE. Normally distributed variables were summarized using means and SD; those that were skewed were expressed as median (interquartile range). The independent sample *t* tests or Mann–Whitney tests were used as appropriate. Pearson correlations were used to assess associations of AGE and oxidation products with (previously reported) eGFR [25]. All tests were two-tailed, with *p* < 0.05 considered as significant.

## 3. Results

Baseline clinical characteristics of the three patient groups are shown in Table 1. Between the DM+PE+ and DM+PE- groups (predefined primary analysis), there were no significant differences in age, duration of diabetes, mean arterial pressure, total cholesterol, LDL cholesterol, triacylglycerols, and microalbumin:creatinine ratio at baseline; but in DM+PE+, BMI, HbA1C, and eGFR were significantly higher, and HDL was significantly lower than in DM+PE-. There were no differences between DM- and DM+PE- groups (predefined secondary analysis) except in HbA1C.

**Figure 1:**
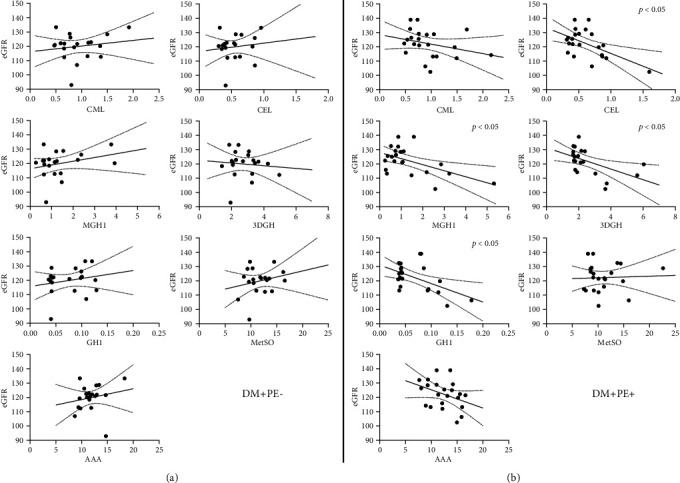
Prospective associations of second trimester (V2) plasma concentrations (nmol/g protein) of five AGE and two oxidation products with third trimester (V3) eGFR (mL/min/1.73 m^2^). Panels show regression lines and 95% confidence intervals. (a) DM+PE-. (b) DM+PE+. In women who subsequently developed PE (b), significant inverse associations with eGFR were observed for four of the five AGE (*p* < 0.05).

Among DM+PE+, cross-sectional inverse associations between AGE and contemporaneous eGFR were observed at both V2 and V3, specifically involving CEL, MGH1, and GH1 at V2 and CML, CEL, MGH1, and GH1 at V3 (Table 2). Prospectively, CEL, MGH1, 3DGH, and GH1 at V2 were inversely associated with eGFR at V3 (all *p* < 0.05), as shown in Figure 1 and Table 3. Notably, there were no significant associations between the two detectable oxidation products and eGFR in diabetic women.

In contrast, among DM+PE- women, there were few associations, only involving CML. CML at V1 was inversely associated with contemporaneous eGFR (Table 2) and prospectively and inversely with eGFR at V3 (not shown) (both *p* < 0.05).

In DM- women, AGE MGH1 and GH1 at V1 were inversely associated with contemporaneous eGFR, while 2-AAA was positively associated at V2 (Table 2). Prospectively, MGH1, GH1, and CEL at V1 were all inversely associated with eGFR at V3 (not shown, *p* < 0.05).

Plasma concentrations of AGE/oxidation-P according to diabetes and PE status are shown in Table 4. Comparing DM+PE+ with DM+PE-, plasma CML, CEL, MGH1, 3DGH, MetSO, and 2-AAA did not differ between these groups at any study visit, while plasma GH1 was marginally lower at V1 in DM+PE+ (*p* < 0.05). Comparing DM+PE- with DM-, despite a trend towards higher values in those with diabetes, very few differences reached significance (*p* < 0.05), and significance was only observed at single gestational time points (2-AAA at V2 and CEL and MGH1 at V3).

sRAGE was measured previously in the whole MAMPED cohort as described [24], and in this study subset was again lower at V2 in DM+PE+ than DM+PE- (*p* < 0.05) (Table 5). Among sRAGE:AGE ratios, only sRAGE:CML and sRAGE:CEL differed according to PE status, both being lower at V2 in DM+PE+ than DM+PE- (*p* = 0.037 and *p* = 0.019, respectively), consistent with prior findings [24]: as before, these effects were driven by differences in sRAGE, not in CML or CEL.

## 4. Discussion

In this study, we investigated whether plasma concentrations of five specific AGE (CML, CEL, GH1, MGH1, and 3DGH) and four oxidation products (MetSO, 2-AAA, 3NT, and DT) were associated cross-sectionally and prospectively with altered renal function (eGFR) and prospectively with late-onset PE in pregnant women with type 1 diabetes. Plasma samples were collected within defined, nonoverlapping, gestational age ranges at each trimester, in all cases in advance of clinical diagnosis of PE.

Considering all diabetic pregnant women as a single group, AGE/oxidation-P were of no predictive value as markers for PE. However, in the second and early third trimesters, diabetic women who subsequently developed PE exhibited inverse associations between four of the five plasma AGE and eGFR (cross-sectionally at both V2 and V3 and prospectively between V2 and V3). In contrast, associations among diabetic women who remained normotensive were negligible. Of the four oxidation products, two were undetectable, and in those that were detectable (MetSO and 2-AAA), there were no associations with eGFR or PE in diabetic women.

Our findings related to eGFR may be germane to the marked variation in susceptibility to vascular complications among diabetic patients, potentially relating to individual variations in susceptibility to AGE-mediated tissue injury. In a previous study, we measured AGE/oxidation-P in skin collagen obtained at biopsy from older, long-duration type 1 diabetic patients recruited as either “prone” or “resistant” to microvascular complications [31]. After accounting for chronologic age, duration of diabetes, and long-term glycemia, we found that complication-prone patients had accumulated higher than expected quantities of these products in collagen, implying reduced antioxidant defenses. Likewise, variation in antioxidant defenses could underpin the present findings. Compared with the earlier skin biopsy study, the current participants were much younger, and the current work addressed concentrations of products in plasma, not in a long-lived tissue protein.

In plasma, AGE and oxidation products are not formed locally (plasma has potent antioxidant properties): they are thought to be derived predominantly from tissues (reviewed, [7]). In long-lived tissues, AGE and oxidation products accumulate slowly with advancing age in everyone; the rate of accumulation varies at least twofold among people without diabetes and is inevitably accelerated by diabetes [32, 33]. In young adults with diabetes, such as those in the present study, the AGE and oxidation product content of long-lived tissues has had less time to diverge from nondiabetic values; this may explain, in part, the similarity between plasma concentrations in the young diabetic vs. nondiabetic pregnant women in this study. As mentioned above, in two other large studies, AGE in plasma were found to predict actual decline in eGFR, but this was over 6-12 years in much older people with more poorly controlled type 2 diabetes [26, 27]. First, in a Pima Indian cohort with type 2 diabetes (at enrollment, mean age 41.4 y; mean HbA1c 9.2% or 77 mmol/mol), a panel of AGE predicted a 40% decline in GFR over six years [26]. Second, and very recently, similar predictive findings were reported in the Action to Control Cardiovascular Risk in Diabetes (ACCORD) study over 12 years' follow-up and in the Veterans Affairs Diabetes Trial over 5 years [27]: in both cases, the study participants were, on average, in the seventh decade of life. Although the present study involves patients who are decades younger, its results are coherent with these findings, suggesting that in people with type 1 diabetes who are predisposed to a complication (in this case, a decline in eGFR), the earliest signs of associations between AGE and renal decline are already evident.

The concepts above are supported by, and consistent with, other recent findings. We recently showed that in cultured placental trophoblast cells, LDL modified by glucose and oxidative stress (i.e., containing AGE/oxidation-P) can potently upregulate expression of antiangiogenic factors (sFlt-1 and soluble endoglin) [34], known to be implicated in the initiation and progression of PE [28, 35]. Excessive circulating levels of these antiangiogenic factors reduce availability of their ligands (VEGF and TGF*β*, respectively) to cell receptors. VEGF and TGF*β* are essential to maintain the normal function of glomerular endothelial cells and glomerular filtration [36]. Secondly, we demonstrated that daily dietary supplementation with polyphenol-rich blueberries and fiber, starting at 18 weeks' gestation in pregnant women at high risk for gestational diabetes, had numerous beneficial effects not only on plasma glucose but also on gestational weight gain, atherogenic lipid subclasses, and particularly, in reducing markers of inflammation (serum C-reactive protein (CRP) and other factors) [37]. These findings are relevant because polyphenols have potent antioxidant activity, and in pregnancy, elevated CRP has been shown by us (in people with diabetes [38]) and by others (in the general population [39]) to be associated with subsequent onset of PE. We also previously showed that dietary (polyphenol) supplementation, again with blueberries, can reduce plasma levels of oxidized LDL, serum “lipoxidation” products (malondialdehyde and hydroxy-nonenal), and systolic and diastolic blood pressure in older patients with metabolic syndrome [40]: all these actions are potentially favorable during pregnancy in reducing risk for PE. Furthermore, we previously reported low serum carotenoid concentrations, indicative of deficient dietary antioxidant status, in women who developed PE compared with those who remained normotensive in our cohort [41]. The present data thus further strengthen the rationale for a safe, dietary antioxidant intervention in diabetic pregnancy: such an approach is promising and important given the immense challenges involved in developing new drugs for use in pregnancy. Unfortunately, we still have limited ability to identify, well in advance, people who are the highest risks for complications of diabetes, but a history of PE appears to serve this role.

AGE accumulation is accelerated not only by the presence of diabetes but also by renal impairment [42]. In MAMPED, we showed that subclinical glomerular injury precedes and predicts the development of PE: eGFR in the first trimester was elevated in women who subsequently developed PE compared with those who did not; furthermore, a significant decrease in eGFR with advancing gestation occurred only in this group [25]. In the present study, among diabetic women, associations were limited to those who later developed PE, suggesting that this group represents a subset of patients who are susceptible to renal damage mediated by AGE. As discussed above, we unfortunately have no data relating to tissue levels or the renal clearance of AGE, but a significant decrease in eGFR across gestation in diabetic women who subsequently developed PE could theoretically result in impaired renal clearance of AGE. Further studies are needed.

This study is the first to provide prospective data on a wide range of AGE/oxidation-P in plasma in diabetic pregnancy. Its strengths include its prospective design, its focus on recruitment of well-characterized diabetic women free of hypertension and albuminuria at baseline, and its rigorous phenotyping of the presence (cases) or complete absence (controls) of PE. The concordance between the present results (using the more rigorous liquid chromatography/mass spectrometry methodology) and our earlier study (using immunologic techniques) lends support to the validity of our findings. Limitations included small study size, the fact that all participants were Caucasian, and the absence of measures of tissue AGE/oxidation-P concentrations in the placenta and kidney. The design of the study does not address causality, nor the role of interventions to reduce AGE-RAGE signaling before or during pregnancy, but is hypothesis-generating.

In conclusion, we show that in pregnant diabetic women, and only among those who developed PE, plasma AGE concentrations in the second and third trimesters were inversely associated with eGFR. AGE may mediate early stages of renal injury in a subset of women who are susceptible to PE, even if they do not identify that subset. Similarly, sensitivity to AGE-mediated tissue damage in women with a history of PE could explain, at least in part, the development of long-term cardiorenal sequelae of PE in diabetes. While measurement of AGE during pregnancy does not aid PE risk stratification, other clinically convenient first trimester markers for PE in type 1 diabetes are emerging [25, 39, 43, 44], allowing identification of patients in whom safe, dietary interventions may be particularly important. Future studies should focus on early markers and mechanisms for PE in diabetes and on the development of safe and rational strategies to prevent both PE and its long-term sequelae.

## Figures and Tables

**Table 1:** Baseline maternal characteristics of women with type 1 diabetes with and without preeclampsia and of normotensive non-diabetic women.

	DM+PE+ (*n* = 23)	*p* value^∗^	DM+PE– (*n* = 24)	*p* value^#^	DM– (*n* = 19)
Age of woman (years)	28.5 ± 5.6	0.31	29.9 ± 3.8	0.25	31.4 ± 4.5
Duration of diabetes (years)	16.8 ± 6.8	0.32	14.8 ± 7.0	—	—
BMI (kg/m^2^)	27.9 ± 5.9	**0.028**	24.6 ± 4.1	0.50	23.8 ± 3.8
HbA_1c_ (%)	7.4 ± 1.2	**0.046**	6.7 ± 1.0	**<0.0001**	5.3 ± 0.3
HbA_1c_ (mmol/mol)	57 ± 14	**0.046**	50 ± 11	**<0.0001**	35 ± 3
MAP (mm Hg)	82.1 ± 9.0	0.21	79.0 ± 7.7	0.14	82.7 ± 6.2
Total cholesterol (mmol/L)	4.7 ± 0.7	0.53	4.5 ± 0.9	0.18	4.9 ± 0.7
HDL cholesterol (mmol/L)	1.9 ± 0.4	**0.029**	2.2 ± 0.5	0.71	2.1 ± 0.6
LDL cholesterol (mmol/L)	2.4 ± 0.7	0.08	2.0 ± 0.7	0.18	2.3 ± 0.8
Triacylglycerols (mmol/L)	1.0 ± 0.3	0.27	0.8 ± 0.3	0.09	1.1 ± 0.4
Microalbumin:creatinine ratio	0.7 (0.5, 1.2)	0.62	0.7 (0.4, 1.0)	0.40	0.8 (0.6, 1.0)
eGFR (mL min^−1^ 1.73 m^−2^)	125.0 ± 7.7	**0.015**	119.4 ± 7.0	0.92	119.7 ± 8.3

Data are presented as means ± SD, or median (interquartile range). Measurements refer to visit 1, at study entry. The independent sample *t* tests and Mann–Whitney tests were used as appropriate. eGFR was defined using Chronic Kidney Disease Epidemiology Collaboration (CKD-EPI) equation. Boldface indicates *p* values < 0.05 (statistically significant). ^∗^*p* value, DM+PE- vs. DM+PE+. ^#^*p* value, DM+PE- vs. DM-.

**Table 2:** Cross-sectional associations (Pearson's correlation coefficients) between plasma concentrations of AGE/oxidation-P and eGFR, during gestation and prior to the onset of preeclampsia.

		DM+PE+		DM+PE-		DM-	
		*R*	*p* value	*R*	*p* value	*R*	*p* value
N^*ε*^-(Carboxymethyl)lysine (CML)	V1	-0.29	0.20	-0.500	**0.018**	0.00	0.99
	V2	-0.30	0.17	0.18	0.43	0.05	0.86
	V3	-0.483	**0.023**	0.06	0.78	0.24	0.33

Carboxyethyl-lysine (CEL)	V1	-0.16	0.48	-0.06	0.80	-0.45	0.07
	V2	-0.463	**0.030**	-0.05	0.82	0.01	0.98
	V3	-0.495	**0.019**	0.04	0.85	-0.01	0.97

Methylglyoxal-hydroimidazolone (MGH1)	V1	0.05	0.84	-0.14	0.54	-0.552	**0.022**
	V2	-0.497	**0.019**	0.22	0.35	0.34	0.17
	V3	-0.429	**0.046**	0.08	0.70	-0.08	0.75

3-Deoxyglucosone hydroimidazolones (3DGH)	V1	-0.12	0.61	-0.12	0.59	-0.40	0.12
	V2	-0.43	0.06	-0.05	0.82	0.34	0.16
	V3	-0.43	0.05	-0.08	0.73	-0.18	0.47

Glyoxal-hydroimidazolone (GH1)	V1	-0.12	0.60	-0.32	0.14	-0.632	**0.006**
	V2	-0.512	**0.015**	0.07	0.78	0.02	0.94
	V3	-0.663	**0.001**	-0.08	0.72	0.14	0.56

Methionine sulfoxide (MetSO)	V1	-0.10	0.67	0.34	0.12	-0.22	0.40
	V2	0.01	0.97	0.26	0.25	0.37	0.13
	V3	-0.38	0.09	-0.02	0.94	0.20	0.42

2-Aminoadipic acid (2-AAA)	V1	0.00	0.99	0.28	0.21	-0.20	0.44
	V2	-0.26	0.24	0.34	0.13	0.552	**0.018**
	V3	-0.41	0.06	0.16	0.46	0.01	0.98

Data are presented as the Pearson correlation coefficient, *p* value. Boldface indicates *p* values < 0.05 (statistically significant). Each AGE product and oxidation product is corrected by total protein concentration. Units for all: nmol/g.

**Table 3:** Prospective associations (Pearson's correlation coefficients) of plasma concentrations of AGE/oxidation-P at the second trimester (V2) with eGFR at the third trimester (before onset of PE in the DM+PE+ group).

	DM+PE+		DM+PE-		DM-	
	*R*	*p* value	*R*	*p* value	*R*	*p* value
CML	-0.291	0.19	0.164	0.49	0.032	0.90
CEL	-0.553	**0.008**	0.124	0.60	-0.047	0.85
MGH1	-0.537	**0.010**	0.281	0.23	0.238	0.34
3DGH	-0.525	**0.017**	-0.092	0.70	0.320	0.20
GH1	-0.528	**0.011**	0.205	0.39	-0.036	0.89
MetSO	0.041	0.86	0.224	0.34	0.112	0.66
2-AAA	-0.364	0.10	0.185	0.44	0.281	0.26

Each AGE product and oxidation product concentration was corrected by total protein concentration. Boldface indicates *p* values < 0.05 (statistically significant).

**Table 4:** Plasma concentrations of circulating advance glycation end products and oxidation products, during gestation and prior to the onset of preeclampsia.

		DM+PE+ (*n* = 23)	*p* value^∗^	DM+PE- (*n* = 24)	*p* value^#^	DM- (*n* = 19)
CML	V1	0.7 ± 0.2	0.45	0.8 ± 0.5	0.42	1.0 ± 0.5
	V2	0.9 ± 0.4	0.91	0.9 ± 0.4	0.10	0.7 ± 0.3
	V3	1.1 ± 0.6	0.45	1.3 ± 0.9	0.39	1.1 ± 0.8

CEL	V1	0.5 ± 0.1	0.33	0.5 ± 0.2	0.93	0.5 ± 0.3
	V2	0.6 ± 0.3	0.38	0.6 ± 0.2	0.36	0.5 ± 0.2
	V3	0.7 ± 0.3	0.92	0.7 ± 0.3	**0.015**	0.5 ± 0.1

MGH1	V1	0.9 ± 0.7	0.22	1.2 ± 0.9	0.36	1.0 ± 0.7
	V2	1.3 ± 1.2	0.63	1.5 ± 1.2	0.24	1.2 ± 0.5
	V3	1.6 ± 1.3	0.25	2.2 ± 2.0	**0.040**	1.2 ± 0.5

3DGH	V1	2.3 ± 1.0	0.20	2.7 ± 1.1	0.69	2.5 ± 2.2
	V2	2.5 ± 1.3	0.29	3.0 ± 1.2	0.07	2.3 ± 1.0
	V3	3.7 ± 1.9	0.72	3.9 ± 2.6	0.14	2.9 ± 1.3

GH1	V1	0.06 ± 0.02	**0.042**	0.08 ± 0.03	0.12	0.06 ± 0.02
	V2	0.07 ± 0.04	0.43	0.08 ± 0.03	0.68	0.07 ± 0.02
	V3	0.10 ± 0.06	0.91	0.10 ± 0.05	0.20	0.09 ± 0.02

MetSO	V1	10.6 ± 7.8	0.92	10.8 ± 2.3	0.24	9.8 ± 2.8
	V2	11.1 ± 3.5	0.60	11.6 ± 2.5	0.10	9.8 ± 3.9
	V3	13.2 ± 7.6	0.82	13.6 ± 5.7	0.15	11.6 ± 2.9

2-AAA	V1	10.7 ± 2.9	0.47	11.4 ± 3.2	0.48	10.8 ± 1.9
	V2	12.4 ± 2.8	0.47	11.8 ± 2.2	**0.024**	9.6 ± 3.5
	V3	13.6 ± 4.8	0.89	13.4 ± 5.2	0.07	10.9 ± 2.5

Data are presented as means ± SD. Each AGE and oxidation product is corrected by total protein concentration. Units for all: nmol/g. Independent sample *t* tests were used as appropriate. Boldface indicates *p* values < 0.05 (statistically significant). ^∗^*p* value, DM+PE- vs. DM+PE+. ^#^*p* value, DM+PE- vs. DM-.

**Table 5:** Plasma concentrations of soluble receptor for advanced glycation end products (sRAGE) and ratios of sRAGE versus advanced glycation end products (AGE), during gestation and prior to the onset of preeclampsia.

		DM+PE+ (*n* = 23)	*p* value^∗^	DM+PE- (*n* = 24)	*p* value^#^	DM- (*n* = 19)
sRAGE^†^	V1	907 ± 324	0.08	1102 ± 405	0.76	1146 ± 473
	V2	942 ± 282	**0.018**	1219 ± 438	0.93	1231 ± 464
	V3	929 ± 465	0.64	984 ± 325	0.58	923 ± 399

sRAGE/CML^‡^	V1	18.2 ± 7.3	0.13	25 ± 18	0.42	21 ± 12
	V2	16.8 ± 7.0	**0.037**	23 ± 11	0.23	28 ± 13
	V3	16 ± 12	0.84	26 ± 8.4	0.84	16.1 ± 8.6

sRAGE/CEL^‡^	V1	30 ± 11	0.37	34 ± 22	0.92	35 ± 13
	V2	27 ± 12	**0.019**	38 ± 17	0.68	40 ± 17
	V3	24 ± 15	0.93	24 ± 11	0.33	28 ± 14

sRAGE/MGH1^‡^	V1	19 ± 10	0.72	21 ± 18	0.73	23 ± 12
	V2	18 ± 14	0.87	19 ± 12	0.96	19 ± 11
	V3	14 ± 12	0.47	11.5 ± 9.8	0.38	14 ± 11

sRAGE/3DGH^‡^	V1	5.9 ± 2.7	0.67	6.2 ± 2.8	0.07	8.2 ± 3.8
	V2	5.9 ± 2.0	0.15	7.0 ± 2.9	0.07	9.1 ± 4.1
	V3	4.8 ± 3.2	0.65	5.2 ± 2.9	0.68	5.6 ± 3.6

sRAGE/GH1^‡^	V1	249 ± 100	0.97	250 ± 157	0.40	288 ± 115
	V2	255 ± 115	0.47	283 ± 133	0.99	283 ± 140
	V3	175 ± 108	0.80	183 ± 90	0.76	175 ± 87

Data are presented as means ± SD. Units: ^†^pg/mL; ^‡^g/moles. Independent sample *t* tests were used as appropriate. Boldface indicates *p* values < 0.05 (statistically significant). ^∗^*p* value, DM+PE- vs. DM+PE+. ^#^*p* value, DM+PE- vs. DM-.

## Data Availability

The data that support the findings of this study are available from the corresponding author on reasonable request.

## Abbreviations

AGE: Advanced glycation end products
CEL: Carboxyethyl-lysine
CML: N^*ε*^-(Carboxymethyl)lysine
CRP: Serum C-reactive protein
DT: Dityrosine
eGFR: Estimated glomerular filtration rate
GH1: Glyoxal-hydroimidazolone
LLOQ: Lower limit of quantitation
MAMPED: Markers and Mechanisms for PreEclampsia in Type 1 Diabetes
MetSO: Methionine sulfoxide
MGH1: Methylglyoxal-hydroimidazolone
PE: Preeclampsia
RAGE: Receptor for advanced glycation end products
sFLT-1: Soluble fms-like tyrosine kinase
VEGF: Vascular endothelial growth factor
2-AAA: 2-Aminoadipic acid
3DGH: 3-Deoxyglucosone hydroimidazolone
3NT: 3-Nitrotyrosine.

## Additional Points

*Guarantor Statement*. TJL has full access to all study data and takes responsibility for the integrity of the data and the accuracy of the data analysis.
